# Danish Holsteins Favor Bull Offspring: Biased Milk Production as a Function of Fetal Sex, and Calving Difficulty

**DOI:** 10.1371/journal.pone.0124051

**Published:** 2015-04-13

**Authors:** Kaare Græsbøll, Carsten Kirkeby, Søren Saxmose Nielsen, Lasse Engbo Christiansen

**Affiliations:** 1 Department of Applied Mathematics and Computer Science, Technical University of Denmark, Lyngby, Denmark; 2 National Veterinary Institute, Technical University of Denmark, Frederiksberg, Denmark; 3 Department of Large Animal Sciences, University of Copenhagen, Frederiksberg, Denmark; Van Andel Institute, UNITED STATES

## Abstract

In a previous study from 2014 it was found that US Holstein cows that gave birth to heifer calves produced more milk than cows having bull calves. We wanted to assess whether this is also true for Danish cattle. Data from 578 Danish Holstein herds were analysed with a mixed effect model and contrary to the findings in the US, we found that cows produced higher volumes of milk if they had a bull calf compared to a heifer calf. We found a significantly higher milk production of 0.28% in the first lactation period for cows giving birth to a bull calf, compared to a heifer calf. This difference was even higher when cows gave birth to another bull calf, so having two bull calves resulted in a difference of 0.52% in milk production compared to any other combination of sex of the offspring. Furthermore, we found that farmer assisted calvings were associated with a higher milk yield. Cows with no farmer assistance or with veterinary assistance during the most recent calving produced less milk. There were also indications that dams would favor a bull fetus by decreasing milk production during the second pregnancy if the calf born in the first parity was a heifer. We hypothesize that size of calves is a confounding factor for milk production. However, calving weight was not available in the present data set to test this hypothesis.

## Introduction

Sex biased milk synthesis is the capability of a mammalian mother to increase or reduce the amount or composition of the milk according to the sex (or sex ratio) of the offspring [1]. Since the growth rate of bull calves is generally higher than for heifer calves [2], it would be expected that a cow would produce more milk to feed a bull calf than a heifer calf. Recently, Hinde *et al.* (2014) [3] documented sex-biased milk production in US Holstein cattle and reported up to 445 kg higher milk production during the two first lactations (≈ 4%) for cows having two heifer calves compared to those having two bull calves. We wanted to assess this result in a comparable Danish data set. Milk production level is a priori the most important parameter in dairy farming, and any favoring of offspring sex may lead to a change in use of sex sorted semen. This could possibly have an economic impact on the milk production, although farmers would also need to secure the supply of heifer calves to sustain the herd.

Calving difficulties can be costly to the farmer if a visit by a veterinarian is required. Calving difficulties can also have a negative impact on the milk yield, which is economically even more serious [4]. Therefore it is important to determine and quantify the impact of calving difficulties in herds.

The objective of this study was to determine if milk yield was affected by the sex of the offspring. Furthermore, we analysed the impact of calving difficulties on the milk yield. We report the results both in kg produced milk and in energy corrected milk yield (*ECM*).

## Materials and Methods

The Danish cattle database, which is managed by SEGES (the knowledge center for agriculture in Denmark, www.seges.dk), stores data on milk production from all milk recorded cattle in Denmark. A random sample of 700 Danish milk recording dairy herds was extracted from the Danish cattle database in November 2013. These herds included 578 farms with Danish Holstein dairy cows of which 71,088 were included in this study. Included dairy cows had registered Danish Holstein calves in their first two calvings, had at least six milk yield records per lactation, and had records on difficulty of calving.

Test-day records from the Danish milk yield recording scheme were obtained from each herd. The milk recording scheme record milk data up to 11 times per herd per year. Milking data were selected so that the second lactation period was completed within 2008 so that sex sorted semen, which was introduced in the middle of 2008, would not influence the results. The earliest data included in the study were from 1997.

To determine the 305 day milk yield, separate Wilmink curves for first and second lactation period [5] including a sinusoidal seasonal effect were fitted per herd, so that the average milk yield per cow per farm could be determined. Milk yield was reported in both kg milk and in energy corrected milk (*ECM*) [6], which is defined as:

$$\begin{array}{c} ECM=milk(0.122\;fat+0.077\;protein+0.249) \end{array}$$ (1)

where *milk* is milk in kilos, *protein* is protein in %, and *fat* is fat in %. Individual measurements from cows were then compared to the average cow on each farm. As an example: a cow is tested ten times during a lactation. However one test is missing, but the remaining test results are on average 10% above the average cow, so the particular cow is estimated to produce on average 10% more than the average cow. In general more than 90% of cows had at least eight samples taken per lactation. All cows with less than five milk samples per lactation were excluded from the data set prior to analysis.

We used a farm based approach to the Wilmink curve as there can be large variations in the average lactation curves between herds. The Wilmink curves were then integrated over time to get the total kg milk or *ECM* yield per farm. The milk yield is then multiplied with the individual cow level to attain the total milk production per cow, *M*.

**Fig 1:**
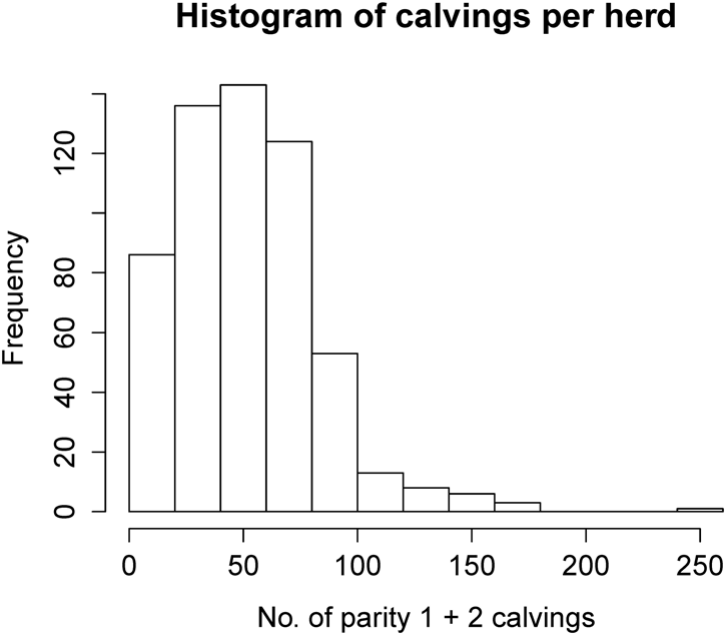
Histogram of calvings per herd. The number of parity 1 and 2 calvings per herd included in the study in the year 2006.

The difficulty of calving is registered in the Danish cattle database by the farmer (see Table 1 for definitions and distribution). The mean number of parity 1 + 2 calvings per herd was 52 in year 2006 (see Fig 1 for the distribution), this year was selected to represent the data as it is the latest year not affected by the right censoring.

**Table 1:** Cross-tabulation of calvings and calving code.

y \ x	1	2	3	4	5	Sum	Description
1	34486	18315	1875	249	27	54952	Easy, without help
2	6106	7663	815	118	24	14726	Easy, with help
3	446	466	100	14	3	1029	Difficult, without veterinary assistance
4	190	143	18	6	0	357	Difficult, with veterinary assistance
5	9	12	1	1	1	24	Cesarian section
Sum	41237	26599	2809	388	55	71088	

Where *x* is the code of first calving and *y* is the code of second calving, *C*_*x*_ *C*_*y*_.

The final model used was a mixed effects model (lme4 version 1.1-7 [7] in R 3.0.2 [8]). Where herds were considered a random effect, while year, difficulty of calving, and sex of offspring were fixed effects:

$$\begin{array}{c} M_{hijk}=\beta_{h}+S_{i}+C_{j}+Y_{k}+S_{i}:C_{j}+\varepsilon \end{array}$$ (2)

where *M* is the total milk yield in kg or *ECM* over a 305 day period. *β*_*h*_ is the random effect of the *h*’th herd. *S*_*i*_ represent the combinations of sex of first and second parity calves. *C*_*j*_ is the fixed effect of the difficulty of calving: factor levels of *j* are 1–5, increasing value indicates increasingly difficult calving: 1 is easy calving with no help, 2 is easy calving with help, 3 is a difficult calving without veterinarian assistance, 4 is a difficult calving with veterinarian assistance, and 5 is calving by cesarian section. Easy calving value 1 is the reference level. *Y*_*k*_ is the fixed effect of the *k*’th year to account for the general improvements in yield over time. The last year, 2008, was chosen as the reference year. *S*_*i*_ : *C*_*j*_ is the interaction term of sex and calving difficulty.

The combinations of sex of first and second parity calves in *S*_*i*_ was expressed as e.g. *B*_1_ *H*_2_ for a bull calf born to a first parity cow and a heifer born in second parity. We first tested a model including the four combinations of heifer and bull calves (*B*_1_ *B*_2_, *B*_1_ *H*_2_, *H*_1_ *B*_2_, *H*_1_ *H*_2_), so that results are directly comparable to the one reported by Hinde *et al*. (2014) [3]. We then reduced the models so that they best fit data. Table 2 shows the distribution of subgroups.

**Table 2:** Cross-tabulation of calvings and sex of calv.

Code \ Sex	*B*_1_ *B*_2_	*H*_1_ *B*_2_	*B*_1_ *H*_2_	*H*_1_ *H*_2_	Sum
*C*_1_ *C*_1_	7669	9424	7724	9669	34486
*C*_2_ *C*_1_	4911	3979	5216	4209	18315
*C*_3_ *C*_1_	650	258	681	286	1875
*C*_4_ *C*_1_	81	41	85	42	249
*C*_5_ *C*_1_	15	1	7	4	27
*C*_1_ *C*_2_	1463	2074	1070	1499	6106
*C*_1_ *C*_3_	128	166	65	87	446
*C*_1_ *C*_4_	59	51	35	45	190
*C*_1_ *C*_5_	1	6	2	0	9
Sum	14977	16000	14885	15841	61703

Notice that this is a subset of Table 1 therefore the sum is lower, and that there is a higher risk of a difficult calving for bull calves.

Odds ratios were tested using Fisher’s exact test in R 3.0.2 [8].

We report the model results as differences between combinations of factors compared to a reference level (direct model output), and transformed back to total production yield. Note that there is additional uncertainty at the overall level which is included when transforming back to the expected yield of cows.

In the study of Hinde *et al*. (2014) [3], data from farms using bovine somatotropin (bST) to increase the milk yield were removed. It is not legal to use bovine somatotropin in the European Union, and therefore this was not a part of our analysis.

## Results

Firstly, the interaction term between sex of offspring and difficulty of calving, *S*_*i*_ : *C*_*j*_, was found to be insignificant (*p* = 0.76). Therefore, all following results refer to the model without interaction.

**Fig 2:**
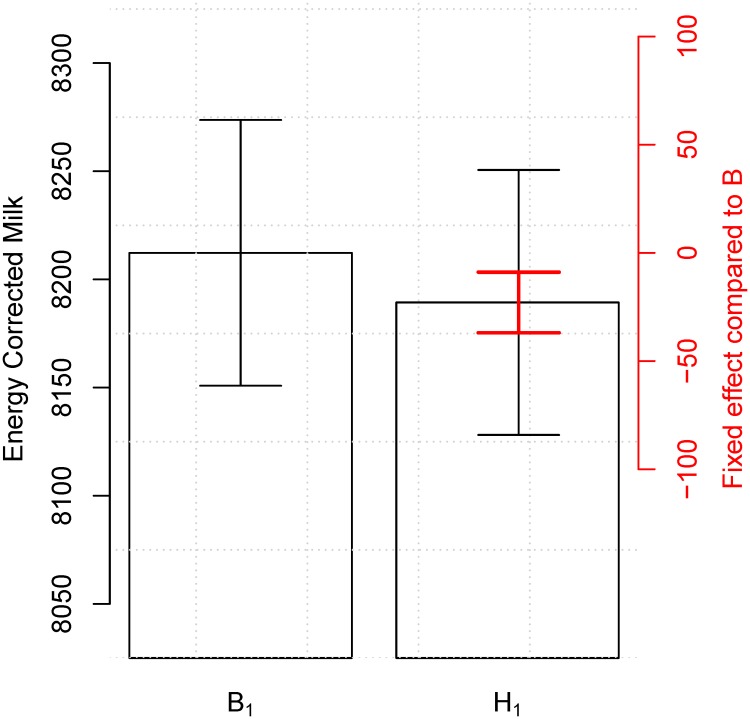
Influence of calf sex on milk production in the first lactation period. When using the best model (Eq 2) including yearly, seasonal, calving, and herd effects there is a significant difference of 22.92 kg *ECM* (*sd* = 7.145) between having a bull or a heifer calf as the first calf (red CI). When including the variance of the reference level the difference is masked (black CI). The numerical results are available in Table 4.

**Fig 3:**
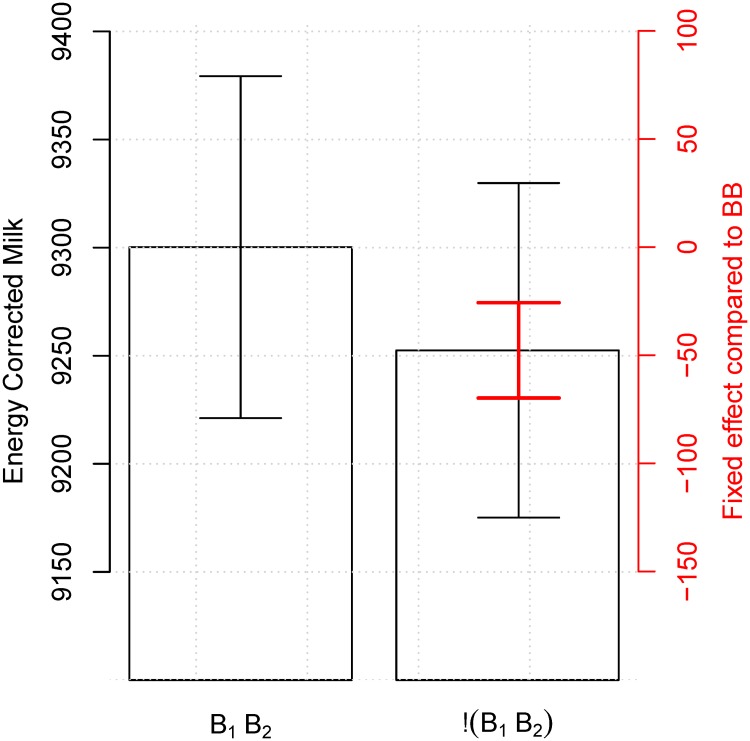
Influence of calf sex on milk production in the second lactation period. When using the best model (Eq 2) including yearly, seasonal, calving, and herd effects there is a significant difference 47.74 *ECM* (*sd* = 11.25) between having two bull calves and any other combination of offspring (red CI). When including the variance of the reference level the difference is masked (black CI). The numerical results are available in Table 6.

**Fig 4:**
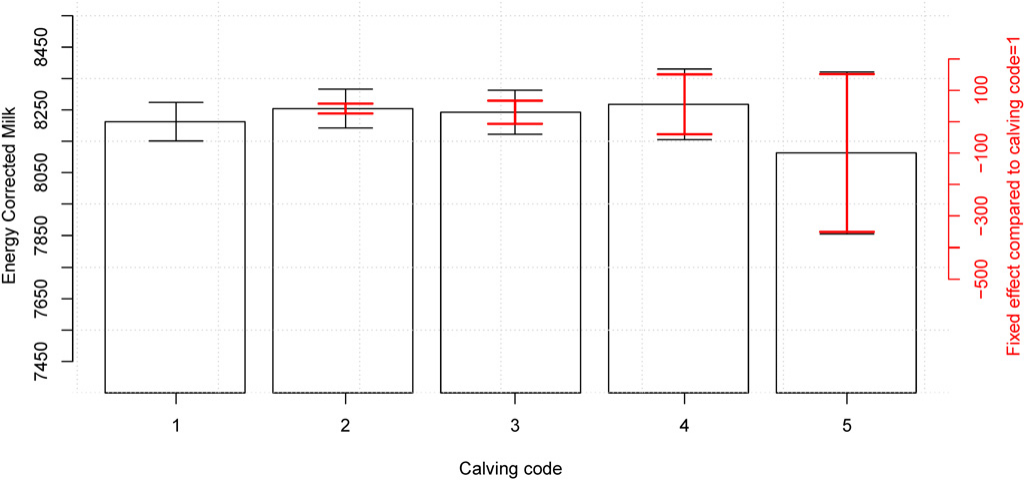
Effect of calving difficulty on milk production in the first lactation period. When using the best model (Eq 2) including yearly, seasonal, calving, and herd effects there is a significant difference of 41.80 *ECM* (*sd* = 8.015) between having an easy calving (calving code = 1) and having a easy calving with help (calving code = 2) (red CI). When including the variance of the reference level the difference is masked (black CI). The numerical results are available in Table 4.

**Fig 5:**
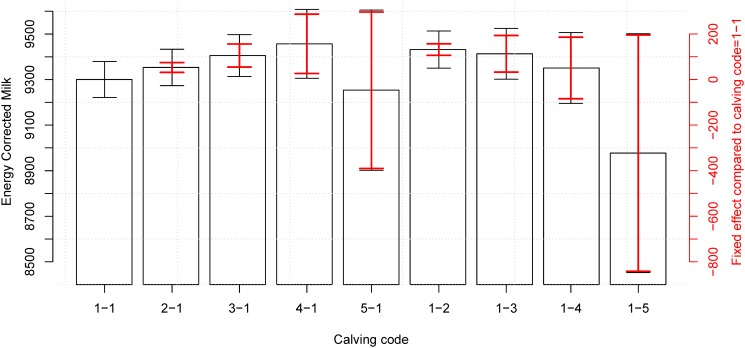
Effect of calving difficulty on milk production in the second lactation period. When using the best model (Eq 2) including yearly, seasonal, calving, and herd effects there is a significant difference of 131.6 *ECM* (*sd* = 12.90) and 113.0 *ECM* (*sd* = 41.09) between having an easy calving in both parities (calving code = 1-1) and more difficult calvings in parity 2 (calving codes = 1-2 and 1-3) (red CI). When including the variance of the reference level there is still significant difference on the total energy corrected milk production (black CI) for calving code 1-2. There is also a significant difference of 53.02, 105.4, and 156.7 *ECM* (*sd* = 10.97, 25.80, 66.46) between having an easy calving in both parities (calving code = 1-1) and more difficult calvings in the first parity (calving codes = 2-1, 3-1, and 4-1 respectively) (red CI). When including the variance of the reference level there is still significant difference on the total energy corrected milk production (black CI) for calving code 3-1. The numerical results are available in Table 6.

We observed for all models tested that milk production was negatively impacted by having heifer calves (Tables 3–6 and Figs 2 and 3); that mildly to moderate calving difficulties improved milk yield (Tables 3–6 and Figs 4 and 5); and that all but two years from the 12 year observation period showed a significant increase in the milk production compared to the previous year (not shown). All results that were significant in *ECM* were also significant in kg milk and vice versa. We present here results of the model including all four combination of sex of offspring during the first two parities so that these can be compared to results presented by Hinde *et al.* [3] (Tables 3 and 5). However, as those models were not the best possible models to represent the data, we also present the best models (Figs 2–5 and Tables 4 and 6).

**Table 3:** Milk production in parity one, all offspring groups.

		kg ECM				kg milk			
Factors		diff	sd	total	sd	diff	sd	total	sd
*B*_1_ *B*_2_	*C*_1_	0	—	8215	31.72	0	—	8255	35.50
*B*_1_ *H*_2_	*C*_1_	-5.292	10.10	8209	31.77	-6.971	10.28	8248	35.55
*H*_1_ *B*_2_	*C*_1_	**-35.13**	9.939	8180	31.62	**-36.46**	10.12	8218	35.41
*H*_1_ *H*_2_	*C*_1_	-15.42	10.06	8199	31.65	-17.62	10.24	8237	35.44
*B*_1_ *B*_2_	*C*_1_	0	—	8215	31.72	0	—	8255	35.50
*B*_1_ *B*_2_	*C*_2_	**41.85**	8.015	8257	31.98	**40.63**	8.160	8295	35.75
*B*_1_ *B*_2_	*C*_3_	30.09	18.83	8245	36.15	30.00	19.17	8285	39.65
*B*_1_ *B*_2_	*C*_4_	55.27	48.66	8270	57.66	54.96	49.53	8309	60.52
*B*_1_ *B*_2_	*C*_5_	-100.1	128.2	8115	131.8	-95.91	130.5	8159	134.9

The impact of sex of offspring, *B*/*H*, and calving difficulty, *C*, on milk yield in kg and *ECM* during the first lactation period, when considering all four possible combinations of sex. The ‘diff’ column is the relative effect compared to the reference indicated by 0; the ‘total’ column is the expected production yield for the given combination of factors; ‘sd’ is the standard deviation. Numbers in boldface are significant on a 95% level.

**Table 4:** Milk production in parity one, best model.

		kg ECM				kg milk			
Factors		diff	sd	total	sd	diff	sd	total	sd
*B*_1_	*C*_1_	0	—	8212	31.34	0	—	8251	35.15
*H*_1_	*C*_1_	**-22.92**	7.145	8189	31.24	**-23.86**	7.272	8227	35.06
*B*_1_	*C*_1_	0	—	8212	31.34	0	—	8251	35.15
*B*_1_	*C*_2_	**41.80**	8.015	8254	31.60	**40.59**	8.160	8292	35.40
*B*_1_	*C*_3_	30.12	18.83	8242	35.83	30.06	19.17	8281	39.35
*B*_1_	*C*_4_	55.47	48.66	8268	57.45	55.15	49.53	8306	60.31
*B*_1_	*C*_5_	-99.41	128.2	8113	131.7	-95.14	130.5	8156	134.9

The impact of sex of offspring, *B*/*H*, and calving difficulty, *C*, on milk yield in kg and *ECM* during the first lactation period, using the best model. The ‘diff’ column is the relative effect compared to the reference indicated by 0; the ‘total’ column is the expected production yield for the given combination of factors; ‘sd’ is the standard deviation. Numbers in boldface are significant on a 95% level.

**Table 5:** Milk production in parity two, all offspring groups.

		kg ECM				kg milk			
Factors		diff	sd	total	sd	diff	sd	total	sd
*B*_1_ *B*_2_	*C*_1_	0	—	9320	39.87	0	—	9357	39.97
*B*_1_ *H*_2_	*C*_1_	**-46.87**	13.60	9273	39.90	**-48.65**	13.75	9308	40.00
*H*_1_ *B*_2_	*C*_1_	**-49.55**	13.30	9270	39.84	**-50.43**	13.45	9306	39.94
*H*_1_ *H*_2_	*C*_1_	**-63.48**	13.47	9256	39.83	**-63.63**	13.62	9293	39.93
*B*_1_ *B*_2_	*C*_1_	0	—	9320	39.87	0	—	9357	39.97
*B*_1_ *B*_2_	*C*_2_	**139.2**	12.63	9459	41.03	**137.6**	12.78	9494	41.15
*B*_1_ *B*_2_	*C*_3_	**132.3**	40.02	9452	55.86	**136.1**	40.47	9493	56.24
*B*_1_ *B*_2_	*C*_4_	57.85	67.68	9377	78.01	63.41	68.44	9420	78.71
*B*_1_ *B*_2_	*C*_5_	-309.4	264.8	9010	267.6	-298.5	267.8	9058	270.5

The impact of sex of offspring, *B*/*H*, and calving difficulty, *C*, on milk yield in kg and *ECM* during the second lactation period, when considering all four possible combinations of sex. The ‘diff’ column is the relative effect compared to the reference indicated by 0; the ‘total’ column is the expected production yield for the given combination of factors; ‘sd’ is the standard deviation. Numbers in boldface are significant on a 95% level.

**Table 6:** Milk production in parity two, best model.

		kg ECM				kg milk			
Factors		diff	sd	total	sd	diff	sd	total	sd
*B*_1_ *B*_2_	*C*_1_ *C*_1_	0	—	9300	40.35	0	—	9338	40.45
!*B*_1_ *B*_2_	*C*_1_ *C*_1_	**-47.74**	11.25	9252	39.47	**-48.83**	11.38	9289	39.55
*B*_1_ *B*_2_	*C*_1_ *C*_1_	0	—	9300	40.35	0	—	9338	40.45
*B*_1_ *B*_2_	*C*_2_ *C*_1_	**53.02**	10.97	9353	40.77	**52.48**	11.10	9391	40.87
*B*_1_ *B*_2_	*C*_3_ *C*_1_	**105.4**	25.80	**9406**	46.91	**102.2**	26.09	**9440**	47.13
*B*_1_ *B*_2_	*C*_4_ *C*_1_	**156.7**	66.46	9457	77.21	**157.8**	67.20	9496	77.89
*B*_1_ *B*_2_	*C*_5_ *C*_1_	-46.75	175.2	9253	179.4	-47.41	177.2	9291	181.3
*B*_1_ *B*_2_	*C*_1_ *C*_2_	**131.6**	12.90	**9432**	41.63	**130.2**	13.04	**9468**	41.75
*B*_1_ *B*_2_	*C*_1_ *C*_3_	**113.0**	41.09	9413	57.01	**116.5**	41.55	9455	57.40
*B*_1_ *B*_2_	*C*_1_ *C*_4_	50.68	69.01	9351	79.40	55.87	69.79	9394	80.11
*B*_1_ *B*_2_	*C*_1_ *C*_5_	-323.1	264.6	8977	267.5	-312.2	267.6	9026	270.4

The impact of sex of offspring, *B*/*H*, and calving difficulty, *C*, on milk yield in kg and *ECM* during the second lactation period, using the best model. The ‘diff’ column is the relative effect compared to the reference indicated by 0; the ‘total’ column is the expected production yield for the given combination of factors; ‘sd’ is the standard deviation. Numbers in boldface are significant on a 95% level.

For the first lactation period, the model with all four combinations of calves showed that cows having two bull calves, *B*_1_ *B*_2_, produced significantly more milk compared to cows in the *H*_1_ *B*_2_ group, but not more than the other groups (top part of Table 3). There was also a marked difference in milk yield between dams carrying a bull or a heifer fetus when the first born was a heifer (*H*_1_ *B*_2_ vs *H*_1_ *H*_2_). However, a model using only the sex of the first offspring was not significantly different from the model including the sex of the second parity calf (*p* = 0.12), therefore the simplest model was chosen. Hence, the best model to describe impact of the sex of the offspring on the milk production in the first lactation period was the model using only the sex of the firstborn calf. This model gave a lower yield of 22.92 *ECM* (*sd* = 7.145) when having a heifer calf compared to a bull calf as first born (Fig 2 and top part of Table 4).

The results presented on sex of offspring is contrasted with an easy calving (calving code = 1) given that this is the most frequent code observed in the data (Table 1).

For the second lactation period the model with all four combination of calves showed that having two bull calves, *B*_1_ *B*_2_, significantly increased milk production compared to the other groups, but there were no significant differences between these other groups (top part of Table 5). Therefore, a model comparing two bull calves, *B*_1_ *B*_2_, to the other three combinations, {*B*_1_ *H*_2_, *H*_1_ *B*_2_, *H*_1_ *H*_2_} = !(*B*_1_ *B*_2_) was tested. The model including all combinations of offspring was not significantly different than the model using only two bull calves versus other combinations (*p* = 0.45), therefore the simplest model was regarded as the best. In the best model having two bull calves gave an increase of 47.74 kg *ECM* (*sd* = 11.25) in the second lactation when compared to any other combination (Fig 3 and top part of Table 6).

The calving code showed a significant impact in the milk production (Tables 4 and 6) for cows that had an ‘easy calving with help’ (category 2, *C*_2_) in both first and second lactation, compared to ‘easy calving with no help’ (category 1, *C*_1_) (see also Figs 4 and 5). In the second lactation there was also a significant increased milk production for cows that had a difficult calving without veterinary assistance (*C*_3_). Furthermore, in parity 2 there was also a significant impact of the difficulty of the first calving. Calving in first parity with codes 2, 3, and 4 had a positive impact on the milk production in the second lactation. Calvings with code 5 (cesarian section) showed a large decrease in milk production, although this was not significant due to a limited number of observation in the class (Table 1). It was not possible to test for interactions between the effect of calving difficulties in first and second calvings due to no records with *C*_5_ in first parity combined with a *C*_4_ in second parity—furthermore only few records exist with high levels of difficulties in both first and second calving.

The random effect of farms revealed a very large variation in the *ECM* between farms (*sd* = 848). There was found strongly significant odds ratios of 1.61 for first parity and 1.43 (both p < 0.001) for bull calves correlating with calving difficulty of 2 or higher (See also Table 2).

## Discussion

We demonstrated that dams produced more milk following the birth of a bull calf. Furthermore, being pregnant with a bull fetus may reduce milk production to possibly increase the energy spent on the bull fetus. Also we demonstrated that a moderate increase in the calving difficulty correlated with a higher milk production.

Before initiating this study we expected to find similar results to Hinde *et al.* (2014) [3], that stated that Holsteins experience a reduction in milk yield when having bull calves. Furthermore, our expectation was that an easier calving would be less exhaustive for the dam, so that it may retain more energy for producing milk, and hence produce a higher amount of milk. Both of these assumptions were not supported by our analysis, which shows that dams’ milk synthesis favor bull calves; and that a moderately difficult calving is correlated with an increase in milk production, probably through some confounding variable.

The Thrivers hypothesis states that dams may invest more (measured in milk production) in male offspring because they profit (in terms of number of offspring) relatively more from investing in bull calves than in heifer calves [9]. This theory presupposes that maternal investment (milk production) will be beneficial into the adulthood of the offspring. This strategy occurs specifically in species with male-male competition in individuals in good condition, in this case meaning that the mating competition between bulls is higher than between cows, and therefore it is better to invest more in bull calves by producing more milk. The Thrivers hypothesis also depicts that investment in female offspring will be more profitable than in male offspring when the individuals are in poor condition because the chance of producing competitive male offspring is low. The milk production is a large part of the maternal investment, and therefore the results found here could indicate that Danish Holstein cattle are generally in good and competitive condition because the dams produce more milk for bull calves. It could possibly also indicate that Holstein cattle in the US are comparable in a worse condition, or of a genetically diverging strain, because they apparently invest more milk in heifer calves. Further research should address this issue, including other cattle populations, and it would be interesting to test this hypothesis by correlating the investment strategy with an animal welfare index.

Dams that were in their first lactation period following a heifer calf and pregnant with a bull fetus, *H*_1_ *B*_2_, produced less milk compared to dams pregnant with a heifer fetus, *H*_1_ *H*_2_, (Table 3), indicating that more energy is used on the bull fetus which is also a possibly favoring mechanism. This difference was only near significant (*p* < 0.1), and we do not observe the same effect when the first born calf is a bull, indicating that dams favor living bull offspring over unborn bull offspring, but unborn bull offspring over living heifer offspring.

For calving difficulties we found that the milk production was increased for cows having a slightly difficult calving (*C*_2_ up to *C*_3_ in present parity). But dams with cesarian section (*C*_5_) produced less than dams with no or little difficulty. Likewise, Eaglen *et al*. (2011) [10] found that moderate difficulties in calving resulted in higher milk production in UK Holstein-Friesian dams. Eaglen *et al*. (2011) [10] speculated that “easy calvings without help” might be wrongly registered so in fact some of them might have had some difficulties without the farmer’s notice. If that is true, the farmer-assisted calvings in category 2 and 3 (where we detected an increase in the milk production during the following lactation) might have had a positive impact in terms of extra care from the farmer, nutrition etc. Furthermore, Eaglen *et al*. (2011)[10] mentions the possibility that more valuable cows (with highly valuated genetic material) may be offered calving assistance from the farmer more often.

A curious result is that a difficult calving of code 2-4 for the first calf may positively affect the milk yield of the second lactation period (Table 6), while only calving code up to 3 have a positive effect on the current lactation period. We hypothesize that this effect is due to stronger cows being more likely to survive to a second lactation, perhaps because farmers may be inclined to only let the cow get inseminated a second time if it produces high amounts of milk, to avoid further difficult births.

We propose that an increase in milk production which is caused by having a bull calf or a difficult calving (Figs 2–5 and Tables 3–6) is an effect of the size of the calf. It has previously been shown that the calving weight of the calf positively influences the milk yield [11–13], and given bull calves are larger than heifer calves [14], and heavier calves more often causes problems during calving [15, 16] this could account for at least some of this correlation. Unfortunately calving weight is not registered routinely in Denmark so this hypothesis could not be tested in this study. We did, however, observe that bull calves are around 50% more likely to cause a difficult birth (calving code higher than one), which could indicate that they are larger at birth. Given that the interaction term on sex and calving difficulty was insignificant, there might be two separate effects on milk yield: one from the sex and one from the birth weight of the calf.

Following the hypothesis of birth weight as a confounder of milk production the disagreement between our results and those of Hinde *et al.* (2014) [3] might be due to a higher emphasis in the US on milk production which may results in selective breeding that causes heifer calves to surpass bull calves in birth size, compared to a more balanced approach to breeding in Denmark [17], but whether this is enough to account for the difference in milk yield need to be verified by further analysis, which should include calving weights of newborn calves and a welfare index.

Overall for Danish farms the difference in milk production due to the sex of the offspring was generally small and smaller than the difference between farms, so it seems that other factors (e.g. management related) are more important for the milk production level. The differences that were identified might be due to size of the offspring rather than the sex, but size and sex might also be separate effects.
